# Palbociclib resistance confers dependence on an FGFR-MAP kinase-mTOR-driven pathway in *KRAS*-mutant non-small cell lung cancer

**DOI:** 10.18632/oncotarget.25803

**Published:** 2018-08-03

**Authors:** Eric Haines, Ting Chen, Naveen Kommajosyula, Zhao Chen, Grit S. Herter-Sprie, Liam Cornell, Kwok-Kin Wong, Geoffrey I. Shapiro

**Affiliations:** ^1^ Department of Medical Oncology, Dana-Farber Cancer Institute, Boston, MA, USA; ^2^ Department of Medicine, Brigham and Women's Hospital and Harvard Medical School, Boston, MA, USA; ^3^ Perlmutter Cancer Center, New York University, Langone Medical Center, New York, New York, USA; ^4^ Novartis Institutes for BioMedical Research, Cambridge, MA, USA; ^5^ University Hospital of Cologne, Weyertal, Cologne, Germany

**Keywords:** CDK4/6 inhibitor, MEK inhibitor, FGFR1, KRAS-mutant non-small cell lung cancer, drug resistance

## Abstract

CDK4 is emerging as a target in *KRAS*-mutant non-small cell lung cancer (NSCLC). We demonstrate that *KRAS*-mutant NSCLC cell lines are initially sensitive to the CDK4/6 inhibitor palbociclib, but readily acquire resistance associated with increased expression of CDK6, D-type cyclins and cyclin E. Resistant cells also demonstrated increased ERK1/2 activity and sensitivity to MEK and ERK inhibitors. Moreover, MEK inhibition reduced the expression and activity of cell cycle proteins mediating palbociclib resistance. In resistant cells, ERK activated mTOR, driven in part by upstream FGFR1 signaling resulting from the extracellular secretion of FGF ligands. A genetically-engineered mouse model of *KRAS*-mutant NSCLC initially sensitive to palbociclib similarly developed acquired resistance with increased expression of cell cycle mediators, ERK1/2 and FGFR1. In this model, resistance was delayed with combined palbociclib and MEK inhibitor treatment. These findings implicate an FGFR1–MAP kinase–mTOR pathway resulting in increased expression of D-cyclins and CDK6 that confers palbociclib resistance and indicate that CDK4/6 inhibition acts to promote MAP kinase dependence.

## INTRODUCTION

*KRAS* mutations occur in approximately 15-30% of non-small cell lung cancers (NSCLCs) [1]. These mutations are associated with a poor response to chemotherapeutics and EGFR targeted therapies, and an overall shortened survival across disease stages [2–5]. The development of effective strategies for this lung cancer subset remains a critical unmet medical need. Although pharmacological targeting of KRAS is being investigated [6], the majority of efforts have focused on the inhibition of downstream components of KRAS-driven pathways. Preclinical modeling has suggested the dominance of the mitogen-activated protein kinase (MAPK) pathway in KRAS-driven cancers, translating to trials of MEK inhibition [7]. However, MEK inhibitors have shown limited clinical success either as monotherapy or in combination with chemotherapy [8, 9].

With the disappointment of MEK inhibitor-based clinical trials, it has become essential to identify novel therapeutic targets in *KRAS*-mutant NSCLC. ERK1/2 activation, downstream of activated KRAS and MEK, is well described for its role in promoting cell cycle progression and proliferation by enhancing the expression of D-cyclins [10] that are critical for promoting the transition from G1 to S phase. D-cyclins form complexes with cyclin-dependent kinases 4 and 6 (CDK4/6), which phosphorylate and contribute to inactivation of the retinoblastoma protein (RB) [11]. In lung cancer cell lines and genetically-engineered mouse models, depletion, genetic ablation or inhibition of CDK4/6 has demonstrated synthetic lethality in *KRAS-*mutant, but not wild-type backgrounds, suggesting the therapeutic relevance of selective CDK4/6 inhibition [12].

In a recent phase 2 trial of the CDK4/6 inhibitor palbociclib in unselected patients with advanced NSCLC, 8/16 patients had stable disease lasting > 4 months [13]. Similarly, in a NSCLC expansion cohort of the phase I trial of abemaciclib, the disease control rate was 55.2% among 29 patients with *KRAS*-mutant tumors; nine remained on abemaciclib 6-25 months [14]. Moreover, CDK4/6 inhibition has been reported to synergize with MEK inhibition in a variety of cancers driven by mutant *RAS*, including NSCLC [15–19].

Here, we demonstrate that the initial efficacy of palbociclib in both *in vitro* and *in vivo* models of *KRAS*-mutant NSCLC is complicated by the rapid onset of acquired resistance, mediated by increased expression of CDK6, cyclin D1, cyclin D3 and cyclin E. Additionally, we establish the importance of increased ERK1/2 activity in palbociclib-resistant cells that mediates D-cyclin and CDK6 expression; ERK activity is controlled upstream in part by FGFR1 and exerts its effects through mTOR activation. Therefore, palbociclib exposure results in MAP kinase pathway dependence, in part explaining the synergism observed between CDK4/6 and MEK inhibition.

## RESULTS

### Cyclin D-CDK6 and cyclin E-CDK2 complexes mediate palbociclib resistance in *KRAS*-mutant NSCLC cells

**Figure 1:**
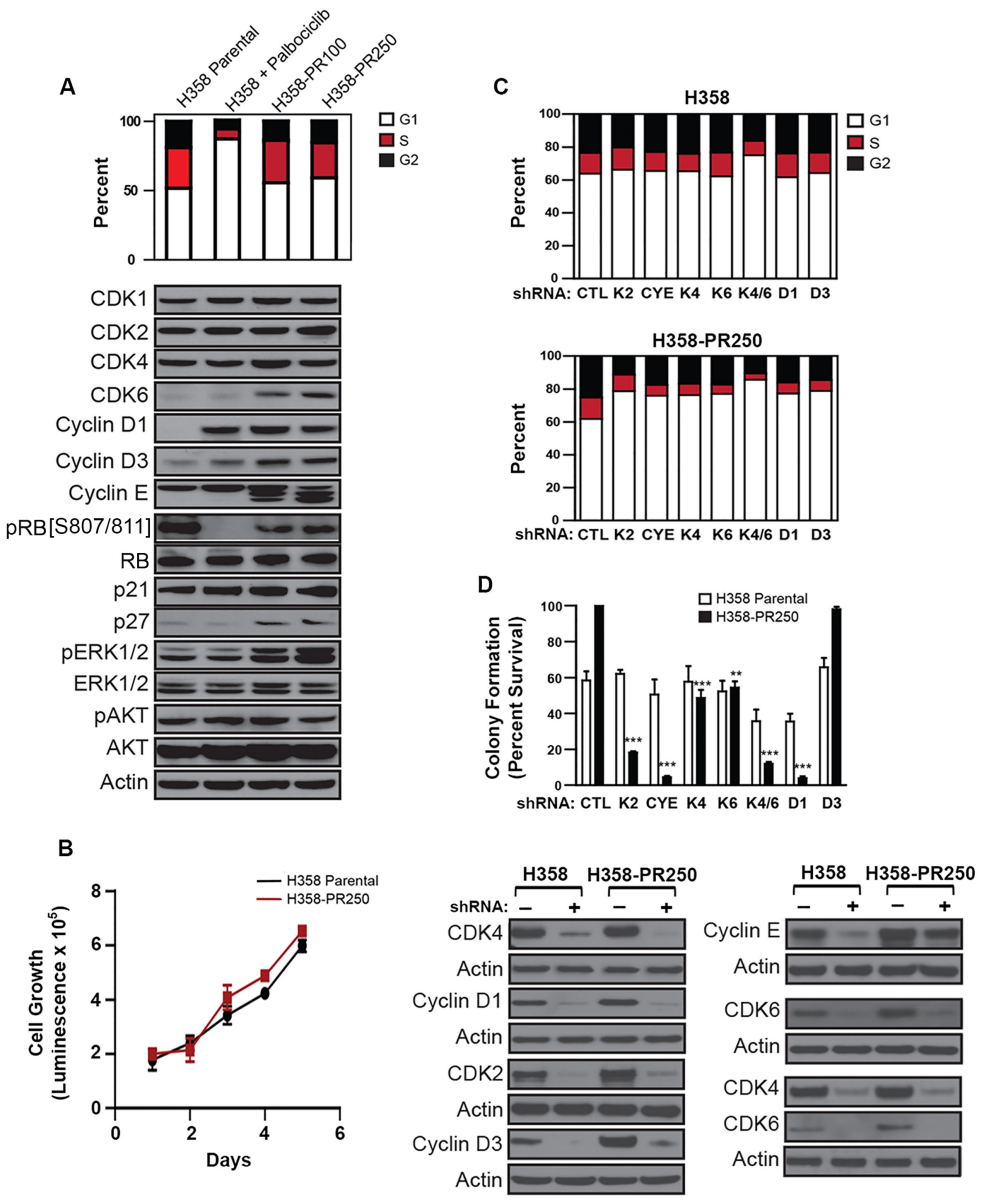
Up-regulation of G1 cyclins and CDKs mediates palbociclib resistance in NSCLC cells **A.** *Upper panel*. Cell cycle profile of H358 cells treated with vehicle control or palbociclib (250 nM) for 24 hours and palbociclib-resistant H358-PR100 and H358-PR250 clones grown in the presence of 100 and 250 nM of palbociclib, respectively. *Lower panel*. Western blot analysis of expression of CDKs and cyclins, as well as expression and activation of ERK1/2 and AKT in parental cells, parental cells treated with 250 nM palbociclib for 24 hours, and palbociclib-resistant H358-PR100 and H358-PR250 cells. **B.** Cell growth of parental H358 cells, in the absence of palbociclib, and H358-PR250 cells, grown in the presence of 250 nM palbociclib assessed by CellTiter Glo assay at the indicated time points. **C.** Cell cycle profiles of untreated parental H358 cells (*upper panel*) and H358-PR250 cells in the presence of palbociclib (*lower panel*) virally infected with the indicated shRNA constructs. **D.** *Upper panel*. Colony formation assay following 14 days of palbociclib treatment (250 nM) of parental H358 cells and H358-PR250 cells infected with the indicated shRNA constructs. Significance was assessed by one-way analysis of variance (ANOVA); ** *P* < 0.01, *** *P* < 0.001. Results represent the average of 3 independent experiments. *Lower panel*. Protein depletions were confirmed by Western blot analysis.

To elucidate mechanisms of acquired resistance to CDK4/6 inhibition, we treated H358 cells with 100 nM palbociclib for 5-6 weeks and isolated a resistant population (H358-PR100; Figure 1A). These cells underwent G1 arrest after exposure to 250 nM palbociclib, but after an additional 4 weeks in culture, a resistant population again emerged (H358-PR250). Whereas treatment of parental H358 cells with palbociclib at 250 nM for 24 hours induced G1 arrest, both palbociclib-resistant cell populations traversed the cell cycle and maintained a similar growth rate to untreated parental cells (Figure 1B). Palbociclib resistance was maintained in the H358-PR250 cells by continuous growth in the presence of palbociclib in all experiments. We similarly derived H441 and H460 cells resistant to palbociclib at doses of 250 and 500 nM, respectively (Supplementary Figure 1A).

Acute exposure of parental H358 cells to palbociclib resulted in increased expression of cyclin D1, as well as a modest increase in cyclin D3, although RB phosphorylation was inhibited (Figure 1A). Cells with acquired resistance to palbociclib demonstrated persistent elevation of cyclin D1, along with increased expression of CDK6, cyclin D3, cyclin E and p27^Kip1^, with restored phosphorylation of RB. Additionally, we observed a novel elevation in ERK1/2 phosphorylation in the resistant cell population. In contrast, the PI3K/AKT pathway was not modulated (Figure 1A). Similar increases in expression of these cell cycle proteins and activation of ERK1/2 were observed in both palbociclib-resistant H441 (H441-PR500) and H460 (H460-PR500) cells (Supplementary Figure 1B).

To confirm the role of G1 cyclins and CDKs in mediating palbociclib resistance in NSCLC cells, we assessed cell cycle arrest after their depletion from both parental and resistant cells (Figure 1C), as well as colony formation after 14 days in 250 nM palbociclib (Figure 1D). The individual depletion of CDK2, CDK4, CDK6, cyclin D1, cyclin D3 and cyclin E had no substantial effect on the cell cycle profile of parental H358 cells cultured in the absence of palbociclib, likely related to compensation by other G1 proteins. Similarly, individual depletion of these proteins did not further compromise colony formation beyond that achieved by palbociclib alone. In contrast, depletion of these proteins in H358-PR250 cells, cultured in the presence of palbociclib, resulted in an increase in G1 content, and with the exception of cyclin D3, reduced colony formation in palbociclib, indicating that their depletion re-sensitized resistant cells. Of note, co-depletion of CDK4 and CDK6 compromised cell cycle progression and colony formation in both parental and resistant H358 cells; however, these effects were more substantial in the resistant population.

We next sought to determine if palbociclib resistance was reversible. We cultured H358-PR250 cells in the absence of palbociclib for a period of 6 weeks (H358-PR250*). The resistant population was re-sensitized to CDK4/6 inhibition after prolonged culture in the absence of palbociclib (Supplementary Figure 2A). Importantly, these re-sensitized cells exhibited reduced CDK6 and cyclin E expression as well as reduced ERK1/2 activation compared to H358-PR250 cells (Supplementary Figure 2B).

### Palbociclib-resistant NSCLC cells are sensitive to MEK and ERK1/2 inhibition

**Figure 2:**
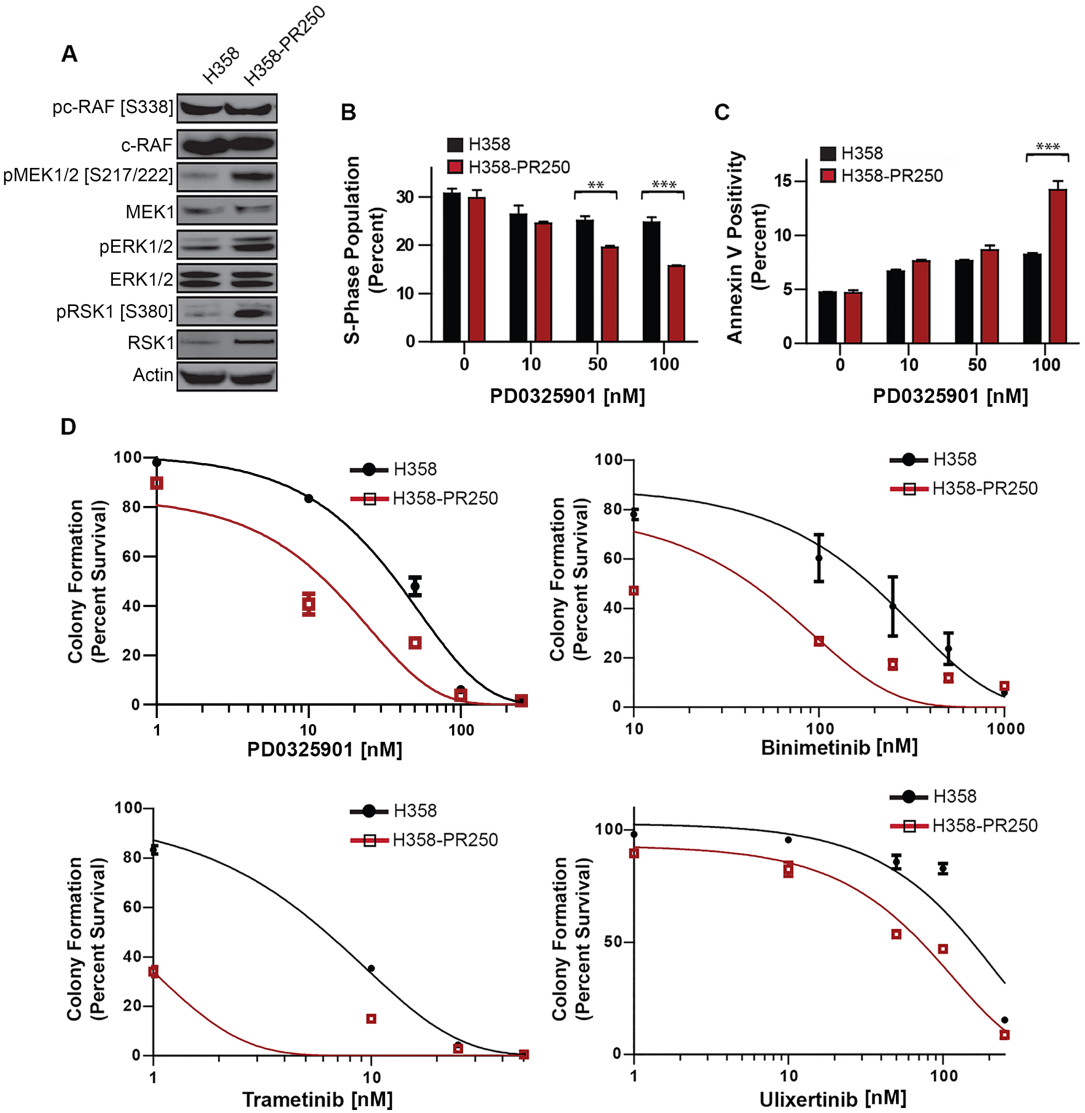
Palbociclib-resistant NSCLC cells are sensitized to MEK/ERK inhibition **A.** Western blot analysis assessing the activity of c-RAF, MEK1/2, ERK1/2 and RSK1 in parental H358 and H358-PR250 cells. **B.** S phase population obtained by flow cytometry of parental H358 cells, grown in the absence of palbociclib, and H358-PR250 cells, grown in the presence of palbociclib, treated for 24 hours with indicated concentrations of PD0325901. **C.** Percent of apoptotic cells, measured by Annexin V staining, in parental H358 cells and H358-PR250 cells, maintained in the absence or presence of palbociclib, respectively, treated with the indicated concentrations of PD0325901 for 72 hours. **D.** Colony formation assays of parental H358 cells and H358-PR250 cells treated 14 days with the indicated concentrations of PD0325901, binimetinib, trametinib and ulixertinib. Significance was assessed using a two-way ANOVA with Bonferroni post-analysis with ** *P* < 0.01, *** *P* < 0.001. Results represent the average of 3 independent experiments.

The enhanced ERK1/2 activity in palbociclib-resistant populations compared to parental H358, H441 and H460 cells (Figure 1A and Supplementary Figure 1B) was likely the result of increased signaling through MEK1/2 (Figure 2A). Additionally, signals downstream of ERK1/2 through RSK1 were also enhanced. To assess the importance of MEK1/2 in mediating palbociclib resistance, we determined the sensitivity of both H358 and H358-PR250 cells to the pharmacological inhibition of MEK. First, we assessed the ability of the MEK inhibitor PD0325901 to induce cell cycle arrest (Figure 2B) or apoptosis, the latter measured by Annexin V positivity (Figure 2C). H358-R250 cells demonstrated a greater degree of G1 arrest and induction of apoptosis compared to parental cells following PD0325901 treatment for 24 and 72 hours, respectively. Additionally, the increased G1 arrest and apoptotic response observed in MEK inhibitor-treated H358-PR250 cells was associated with a reduction in colony formation (Figure 2D, Supplementary Table 1). The increased sensitivity of palbociclib-resistant compared to parental cells to PD0325901 was also observed in palbociclib-resistant H441 and H460 cells (Supplementary Figure 3). Similar findings were demonstrated with two other MEK inhibitors, binimetinib and trametinib, as well as with the ERK1/2 inhibitor ulixertinib (Figure 2D).

### ERK1/2-dependent mTOR activation promotes palbociclib resistance in NSCLC cells

**Figure 3:**
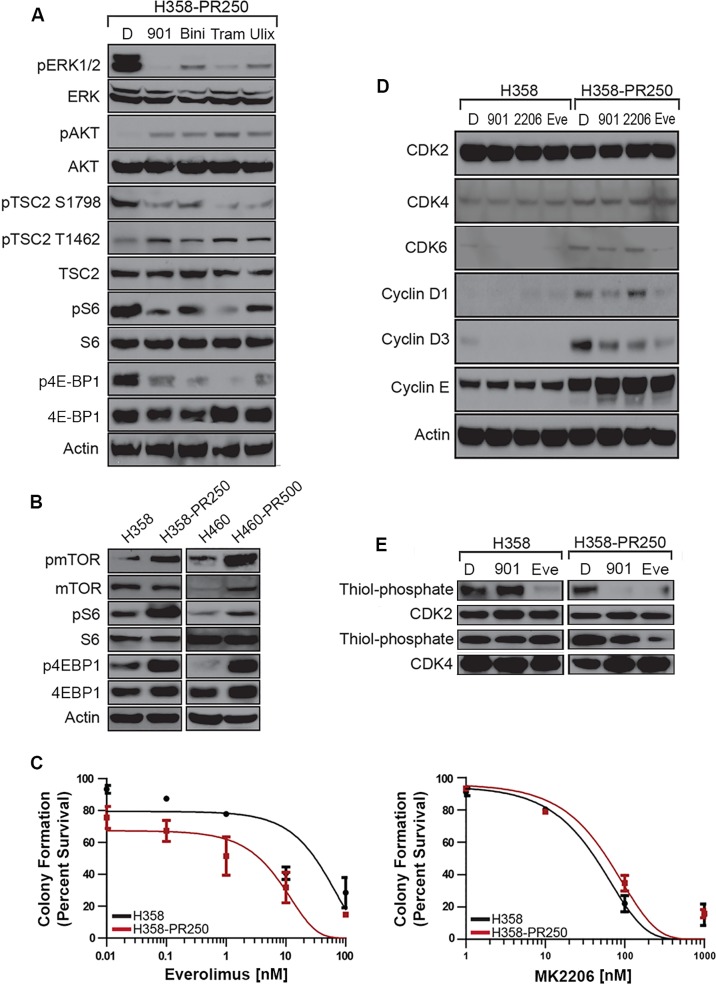
ERK1/2 promotes palbociclib resistance through the activation of the mTOR pathway **A.** Western blot analysis assessing the activation of signaling cascades in H358-PR250 cells treated with DMSO (D), 100 nM PD0325901 (901), 2000 nM binimetinib (Bini), 100 nM trametinib (Tram) or 1000 nM ulixertinib (Ulix) for 24 hours. **B.** Western blot analysis assessing the activity of mTOR, S6 ribosomal protein, 4E-BP1 and TSC2 in parental H358 and H358-PR250 cells (*left panel*) or H460 and H460-PR500 cells (*right panel*). **C.** Colony formation assay assessing the sensitivity of parental H358 and H358-PR250 cells to treatment for 14 days with the indicated doses of everolimus (*left panel*) and MK2206 (*right panel*). **D.** Assessment of CDK and G1 cyclin expression by Western blot analysis in parental H358 and H358-PR250 cells treated with DMSO (D), 100 nM PD0325901 (901), 2000 nM MK2206 (2206) or 100 nM everolimus (Eve) for 24 hours. **E.** Kinase activity assay examining the activity of CDK2 and CDK4 in parental H358 and H358-PR250 cells treated with DMSO (D), 100 nM PD0325901 (901) or 100 nM everolimus (Eve) for 24 hours.

Next, we asked which signaling pathways were modulated by the increased ERK1/2 activity observed in H358-PR250 cells. As shown in Figure 3A, treatment with PD0325901, binimetinib, trametinib or ulixertinib all substantially lowered ERK1/2 activity, correlating with a decrease in ERK1/2-dependent phosphorylation of tuberous sclerosis 2 (TSC2) on Ser^1798^. In contrast, no decrease in AKT-dependent phosphorylation of TSC2 on Thr^1462^ was observed. In fact, we observed increased phosphorylation of AKT and TSC2 at the AKT phosphorylation site suggesting that ERK1/2 may be modulating the activity of the mTOR pathway. Indeed, compared to parental cells, resistant cells demonstrated increased mTOR activity, as measured by mTOR phosphorylation and activation of downstream signaling mediators, including S6 ribosomal protein (S6) and eukaryotic translation initiation factor 4E-binding protein 1 (4E-BP1). Moreover, MEK/ERK inhibition diminished mTOR-dependent signaling with reduced phosphorylation of both S6 and 4E-BP1. Similar results were observed in palbociclib-resistant H460 cells (H460-PR500) (Figure 3B).

We then examined the sensitivity of H358 and H358-PR250 cells to the mTOR inhibitor everolimus and demonstrated an increased sensitivity of the palbociclib-resistant population by colony formation assay (Figure 3C, left panel, and Supplementary Table 1). In contrast, H358PR250 cells did not display increased sensitivity to the AKT inhibitor MK2206, suggesting activation of mTOR by an AKT-independent mechanism (Figure 3C, right panel and Supplementary Table 1). Instead, mTOR activation is ERK1/2-dependent, since ERK1/2 is governing the phosphorylation of TSC2, a key regulator of mTOR activity (Figure 3A).

We sought to link the MEK-ERK1/2-mTOR pathway to the increased expression of G1 cyclins and CDKs that conferred palbociclib resistance. PD0325901 reduced expression of CDK6, cyclin D1 and cyclin D3, but not cyclin E in resistant cells. A similar pattern was observed with everolimus to an even greater extent. In contrast, treatment with the AKT inhibitor MK2206 did not result in decreases in expression of CDK6, cyclin D1 or cyclin E; additionally, only a modest reduction in cyclin D3 expression was observed (Figure 3D).

Since MEK and mTOR inhibition were shown to have no effect on the expression of CDK2, CDK4 and Cyclin E, we subsequently assessed the kinase activity of CDK2 and CDK4 in both parental H358 and H358-PR250 cells treated with either PD0325901 or everolimus (Figure 3E). In parental H358 cells, PD0325901 treatment was shown to have no effect on the kinase activity of CDK2 and CDK4, whereas, everolimus treatment was associated with reduced CDK2 but not CDK4 activity. In contrast, in H358-PR250 cells, treatment with either PD0325901 or everolimus was shown to reduce the kinase activity of both G1 CDKs.

### The interplay of cyclin D-CDK4/6 and cyclin E-CDK2 activity is modulated by p27^Kip1^ in palbociclib-resistant NSCLC cells

**Figure 4:**
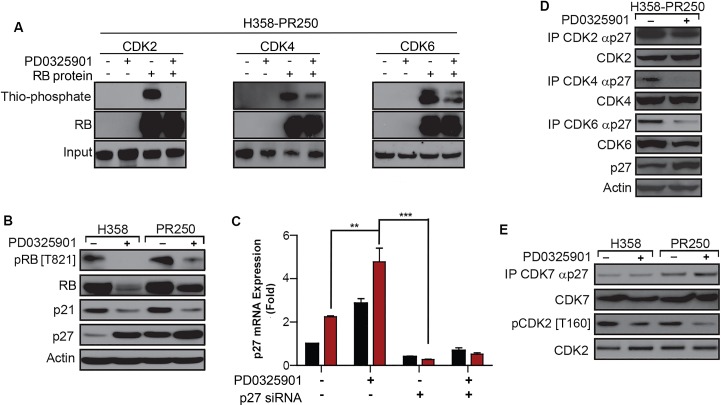
PD0325901 down-regulation of CDK2 activation in palbociclib-resistant cells is dependent on p27^Kip1^ **A.** Kinase activity of CDK2 (*left panel*), CDK4 (*middle panel*) and CDK6 (*right panel*) in H358-PR250 cells treated with vehicle (DMSO) or 100 nM PD0325901 in the absence or presence of RB protein substrate. **B.** Western blot analysis assessing RB phosphorylation and the expression of p21^Waf1/Cip1^ and p27^Kip1^ in parental H358 and H358-PR250 cells treated with vehicle (-) or 100 nM PD0325901 (+) for 24 hours. **C.** p27^Kip1^ mRNA expression assessed by RT-PCR in parental H358 and H358-PR250 cells treated with vehicle or 100 nM PD0325901 without or with siRNA-mediated depletion of p27^Kip1^. Drug treatments were for 24 hours. **D.** Co-immunoprecipitation of p27^Kip1^ with CDK2, CDK4 or CDK6 in H358-PR250 cells treated with vehicle or 100 nM PD0325901 for 24 hours. **E.** Coimmunoprecipitation of p27^Kip1^ with CDK7 in parental H358 and H358-PR250 cells treated with vehicle or 100 nM PD0325901 for 24 hours. CDK7-dependent phosphorylation of CDK2 was also assessed using a phospho-specific antibody to phospho-CDK2 [T160]. Significance was assessed using a two-way ANOVA with Bonferroni post-analysis with ** *P* < 0.01, *** *P* < 0.001; Results represent the average of 3 independent experiments.

The reduction in CDK6 and D-cyclin expression mediated by MEK inhibition in palbociclib-resistant cells explains reduced CDK4 activity (Figure 3E) as well as reduced CDK6 activity (Figure 4A). Although MEK inhibition does not reduce levels of cyclin E (Figure 3D), it is possible that CDK2 activity is modulated by increases in p27^Kip1^. Consistent with this hypothesis, reduced CDK2-dependent RB phosphorylation at Thr^821^ [20] correlated with increased p27^Kip1^ expression in palbociclib-resistant H358 cells treated with PD0325901. MEK inhibition was also associated with a decrease in total RB protein level (Figure 4B). The MEK inhibitor-dependent induction of p27^Kip1^ expression was shown to be mediated at the transcriptional level (Figure 4C).

We next determined whether the modulated activity of CDK2, CDK4 and CDK6 observed in palbociclib-resistant cells upon MEK inhibition correlated with altered associations of these CDKs with p27^Kip1^(Figure 4D). Interactions between CDK4 and p27^Kip1^ were completely abolished in PD0325901-treated H358-PR250 cells. Additionally, the MEK inhibitor-dependent reduction in CDK6 prevented CDK6-p27^Kip1^ interactions. In contrast, only a modest decrease in the association of CDK2 with p27^Kip1^ was observed. To further delineate the actions of MEK inhibition on CDK2 kinase activity, we asked whether PD0325901 potentiated CDK7-p27^Kip1^ complex formation and thus blocked CDK activating kinase (CAK)-dependent activation of CDK2 [21] (Figure 4E). Indeed, both an increased association between CDK7 and p27^Kip1^ and a decrease in activating CDK2 phosphorylation at Thr^160^ were observed in H358-PR250 cells in response to MEK inhibitor treatment.

In summary, our data indicate that palbociclib-resistant cells up-regulate an ERK1/2-mTOR pathway resulting in increased expression of CDK6 and D-cyclins, assembly of which may be facilitated by a concomitant increase in p27^Kip1^ (Figure 1A). Cyclin E expression is also increased in palbociclib-resistant cells. MEK inhibition in palbociclib-resistant cells reduces expression of CDK6 and D-cyclins and further increases expression of p27^Kip1^. After MEK inhibitor treatment, the association of p27^Kip1^ with CDK4/6, where it is needed for cyclin D-CDK4/6 holoenzyme assembly, is decreased. In contrast, p27^Kip1^ association with CDK2, where it is expected to negatively regulate cyclin E-CDK2 activity [22], is maintained, and association with CDK7 is increased, preventing CDK2 activation by CAK, serving to reduce cyclin E-CDK2 activity after MEK inhibition. These events translate to restored G1 arrest, increased apoptosis and reduced colony formation by MEK inhibition in palbociclib-resistant cells.

### Up-regulation of FGFR1 activity mediates ERK-dependent mTOR activation in palbociclib-resistant cells

**Figure 5:**
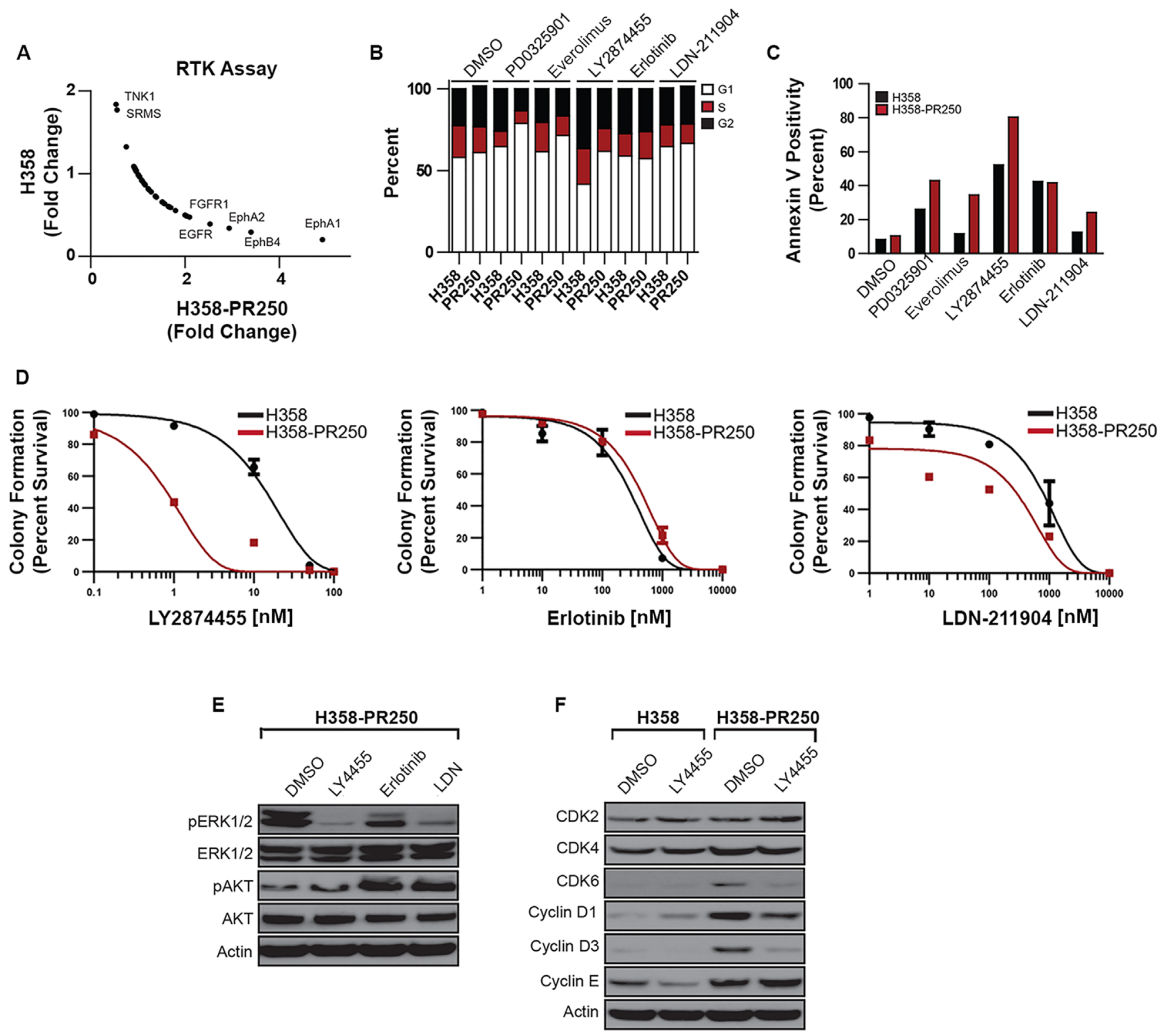
FGFR1 activity promotes signals through ERK/mTor in palbociclib-resistant NSCLC cells **A.** Fold change in the activity of a subset of tyrosine kinases obtained from a tyrosine kinase activity array in parental H358 cells grown in the absence of palbociclib and H358-PR250 cells continuously grown in palbociclib. **B.** Cell cycle analysis of parental H358 and H358-PR250 cells following treatment with 100 nM PD0325901, 100 nM everolimus, 10 nM LY2874455, 10 μM erlotinib, or 2 μM LDN-211904 for 24 hours. **C.** Assessment of apoptosis by Annexin V staining of H358 and H358-PR250 cells treated as in (B) for 3 days. **D.** Colony formation assay assessing the sensitivity of H358 and H358-PR250 to LY2874455 (*left panel*), erlotinib (*middle panel*) and LDN-211904 (*right panel*) at the indicated doses for 14 days. **E.** Assessment of ERK1/2 activity by Western blot analysis of lysates obtained from H358-PR250 cells treated with DMSO or LY2874455 (LY4455), erlotinib or LDN-211904 (LDN) as in (B) for 24 hours. **F.** Assessment of CDK2, CDK4, CDK6, Cyclin D1 and Cyclin D3 expression in parental H358 and H358-PR250 cells treated with DMSO or 10 nM LY2874455 (LY4455) for 24 hours.

Utilizing a kinase activity array, we next sought to determine the kinases involved in mediating the activity of both the ERK1/2 and mTOR pathways. As shown in Figure 5A, H358-PR250 cells demonstrated increased activation of a subset of kinases compared to parental cells, including erythropoietin-producing human hepatocellular receptors A1 and A2 (EphA1/2), epidermal growth factor receptor (EGFR) and fibroblast growth factor receptor 1 (FGFR1). In contrast, activation of two members of the Src kinase family, tyrosine kinase non-receptor 1 (TNK1) and Src-Related Kinase Lacking C-Terminal Regulatory Tyrosine [23, 24] were reduced in these cells.

We next examined the sensitivity of H358 and H358-PR250 cells to inhibitors of these identified kinases, including the pan-Eph receptor inhibitor LDN-211904, the EGFR inhibitor erlotinib and the FGFR1/2 inhibitor LY2874455, by cell cycle analysis, Annexin V staining and colony formation assay (Figure 5B, 5C and 5D; Supplementary Table 1). Compared to parental cells, H358-PR250 cells demonstrated the greatest increase in sensitivity to the FGFR1/2 inhibitor LY2874455 with a modest reduction in S phase DNA content, increased Annexin V positivity, and substantially reduced colony formation. In contrast, erlotinib treatment exerted similar effects in both the EGFR tyrosine kinase inhibitor-sensitive parental H358 cells [25], as well as in H358-PR250 cells. H358-PR250 cells also demonstrated increased sensitivity to LDN-211904 measured by increased Annexin V positivity and reduced colony formation compared to parental cells; however, effects on cell cycle were similar in palbociclib-resistant and parental cells. Overall, the effects of LDN-211904 were less marked than those induced by LY2874455.

To determine whether these kinases mediated the downstream activity of the ERK1/2 pathway in H358-PR250 cells, we treated cells with the pharmacological inhibitors and assessed ERK1/2 activation by Western blot (Figure 5E). The inhibition of FGFR, EGFR or EphRs resulted in decreased ERK1/2 activity; the effects of erlotinib were more modest compared to those mediated by LY2874455 and LDN-211904. Additionally, both erlotinib and LDN-211904, but not LY2874455, were associated with increased AKT activation. Based on the reduction in phospho-ERK and the pronounced biological effects of LY2874455, we focused on further characterizing the role of FGFR1/2 in mediating palbociclib resistance by assessing the expression of G1 CDK and cyclins in response to FGFR1/2 inhibition in both parental and palbociclib-resistant H358 cells. LY2874455 treatment was associated with a decrease in the expression of CDK6, cyclin D1 and cyclin D3 in H358-PR250 cells, but not in parental cells (Figure 5F). These data indicate that FGFR1/2 inhibition most closely phenocopies the effects of MEK and mTOR inhibition in palbociclib-resistant cells.

### Secretion of FGF ligands from resistant cells mediates palbociclib resistance

**Figure 6:**
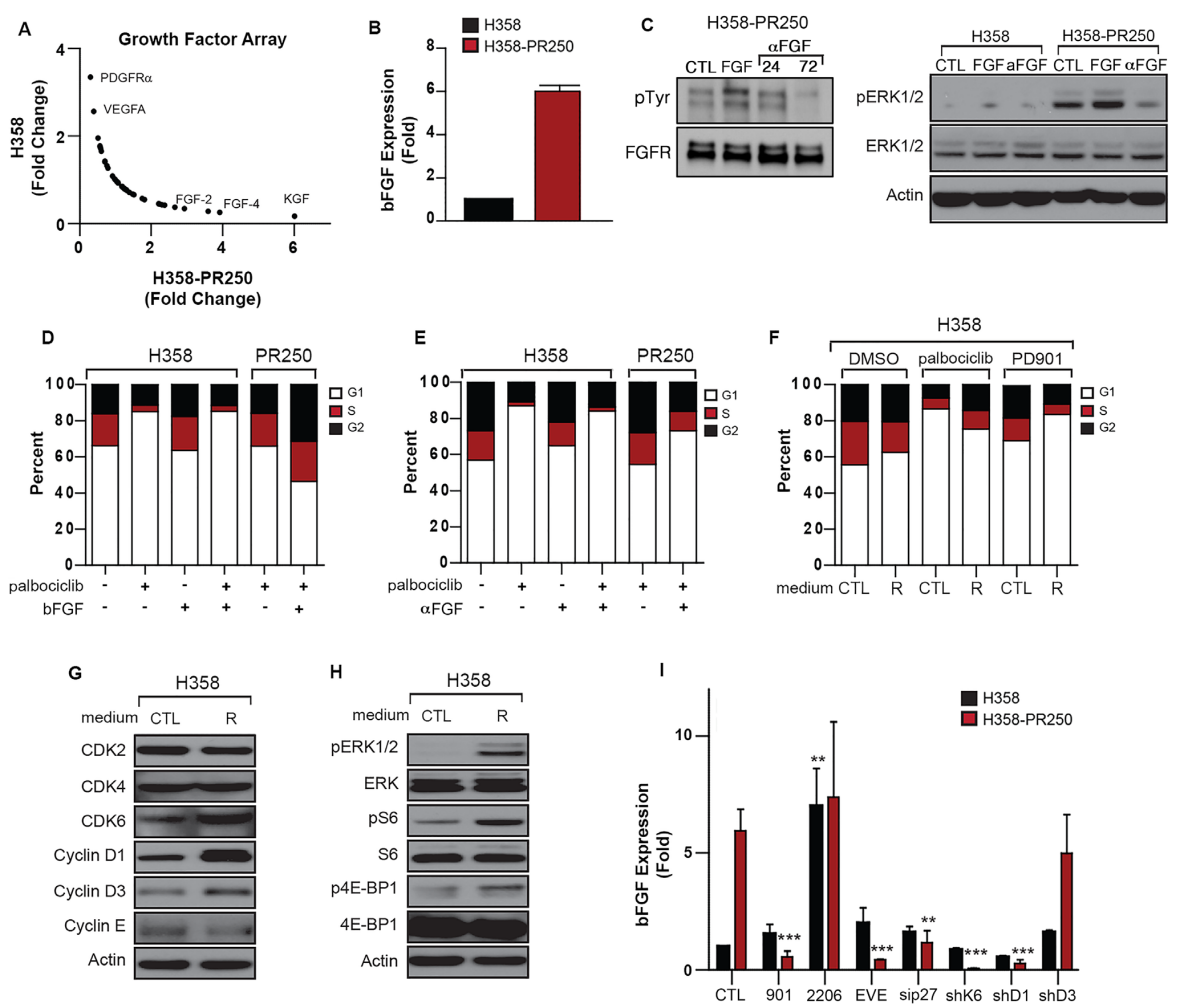
bFGF secretion by palbociclib-resistant NSCLC cells promotes resistance **A.** Fold change in the secretion of growth factors by parental H358 cells grown in the absence of palbociclib and H358-PR250 cells continuously grown in palbociclib from a growth factor array. **B.** mRNA expression of bFGF in parental H358 and H358-PR250 cells assessed by quantitative RT-PCR. **C.** (*Left panel*) Lysates from H358-PR250 cells left untreated (CTL), treated with bFGF ligand for 72 hours (FGF), or a neutralizing antibody to bFGF (αFGF) for 24 or 72 hours were subjected to immunoprecipitation with an anti-FGFR1 antibody followed by Western blot analysis for phospho-tyrosine or FGFR1. (*Right panel*) Western blot analysis assessing the activity of ERK1/2 in parental H358 and H358-PR250 cells left untreated (CTL) or treated with bFGF (FGF) or a neutralizing antibody to bFGF (αFGF) for 72 hours. **D.** Cell cycle profile of parental H358 cells left untreated, treated with palbociclib alone, bFGF ligand for 72 hours alone or in combination with palbociclib (250 nM) for 24 hours; and H358-PR250 cells maintained in palbociclib treated without or with bFGF ligand for 72 hours. **E.** Cell cycle profile of parental H358 cells left untreated, treated with palbociclib alone, a bFGF neutralizing antibody (1 mg/ml) alone for 72 hours, or in combination with palbociclib (250 nM) for 24 hours; and H358-PR250 cells maintained in palbociclib and treated without or with a bFGF neutralizing antibody (1 mg/ml) for 72 hours. **F.** Cell cycle profile of H358 cells grown in medium isolated from parental cells (CTL) or resistant cells (R) for 72 hours then treated with DMSO, palbociclib (250 nM) or PD0325901 (50 nM) for 24 hours. **G.** Western blot analysis assessing the expression of CDKs and cyclins in lysates obtained from H358 cells grown in control (CTL) medium or medium isolated from palbociclib-resistant cells (R) for 72 hours. **H.** Western blot analysis assessing the activation of ERK1/2 and mTOR signaling in parental H358 cells grown in control (CTL) or medium isolated from palbociclib-resistant cells (R) for 72 hours. **I.** bFGF mRNA levels assessed by RT-qPCR in parental H358 and H358PR250 cells left untreated or treated with 100 nM PD0325901, 2 μM MK2206 or 100 nM everolimus for 24 hours; or depleted of p27^Kip1^ by siRNA or CDK6, cyclin D1 or cyclin D3 by shRNA for 72 hours.

To determine what was promoting the activity of FGFR1 in the palbociclib-resistant population, we examined the concentration of growth factors secreted into the medium of parental and resistant H358 cells using a human growth factor array (Figure 6A). The secretion of two growth factors, bFGF (FGF-2) and FGF-4, known activators of FGFR1, was up-regulated in the resistant cell population compared to parental cells. Additionally, keratinocyte growth factor (KGF or FGF-7), an activator of the FGFR2b receptor, was also elevated in the medium of resistant cells. Conversely, we observed a decrease in the angiogenesis-inducing factors VEGFA and PDGFR secreted from the resistant cell population. The increased expression of bFGF was confirmed by quantitative RT-PCR (Figure 6B). H358-PR250 cells were shown to have a 6-fold increase in bFGF transcript levels compared to parental cells. Similarly, in H460-PR500 cells, we observed increased secretion of bFGF, FGF6 and KGF compared with parental H460 cells, whereas, secretion of neurotrophins 4 (NT4) and PDGFR was decreased in resistant cells (Supplementary Figure 4).

To determine whether the secretion of bFGF promoted palbociclib resistance and MEK inhibitor sensitivity, we first assessed the ability of bFGF treatment to further enhance FGFR1 activity and a bFGF neutralizing antibody to block the activation of FGFR1 (Figure 6C, left panel). To do this, we immunoprecipitated total FGFR1 from H358-PR250 cells and detected activated FGFR1 using an antibody against phosphorylated tyrosine residues. Acute treatment with the bFGF ligand (15 minutes) was associated with increased FGFR1 activation, whereas treatment with the bFGF neutralizing antibody significantly blocked FGFR1 activation at 72 hours but not at 24 hours. Next, we assessed the activation of ERK1/2 in parental and resistant H358 cells upon stimulation of FGFR by bFGF treatment or blockade of FGFR using the bFGF neutralizing antibody (Figure 6C, right panel). In the palbociclib-resistant cell population, bFGF stimulation modestly increased ERK1/2 phosphorylation, whereas the neutralizing antibody was effective in reducing ERK1/2 activity.

Next, we assessed the sensitivity of parental and resistant H358 cells to palbociclib following 72 hours of bFGF stimulation (Figure 6D). In H358 parental cells, stimulation with bFGF (50 ng/ml) for 24 hours had no effect on the cell cycle profile in the absence or presence of palbociclib, so that treatment with bFGF was unable to reverse G1 arrest induced by palbociclib. In contrast, in H358-PR250 cells, bFGF stimulation was associated with cell cycle progression, as shown by a decrease in G1 and an increase in S/G2 populations. In parallel, we treated both parental and palbociclib-resistant cells with the bFGF neutralizing antibody for 72 hours and assessed the sensitivity of cells to palbociclib (Figure 6E). Treatment of parental H358 cells with the neutralizing antibody slightly increased the G1 population, although a similar G1 arrest was observed in cells treated with palbociclib alone or in combination with the bFGF neutralizing antibody. Importantly, a more substantial G1 arrest was observed in H358-PR250 cells treated with the neutralizing antibody, suggesting a reversal of palbociclib resistance.

We next asked whether culturing parental H358 cells in medium isolated from resistant cells could alter the sensitivity of parental cells to both palbociclib and MEK inhibition. As shown in Figure 6F, when parental H358 cells were cultured for 72 hours in medium isolated from parental cells, we observed an induction of G1 arrest in response to both palbociclib (250 nM) and PD0325901 (50 nM) for 24 hours. In contrast, there was decreased sensitivity to palbociclib when parental H358 cells were cultured in medium isolated from palbociclib-resistant cells. Similar to H358-PR250 cells, parental H358 cells cultured in resistant medium demonstrated increased sensitivity to MEK inhibition (Figure 6F), elevated expression of CDK6, cyclin D1 and cyclin D3 (Figure 6G) and increased signaling through ERK1/2, S6 and 4E-BP1 (Figure 6H). These results were confirmed in other *KRAS*-mutant NSCLC cell lines, including A427, SKLU-1 and H2030. As shown in Supplementary Figure 5, treatment of conditioned media isolated from H358-PR250 cells had no effect on the basal cell cycle profile of these three cell lines. Nonetheless, increased ERK1/2 activity in both H2030 and A427 cells was observed, correlating with increased MEK inhibitor sensitivity in these cell lines. In SKLU-1 cells, the conditioned media modestly enhanced signaling through ERK1/2, although these cells were substantially sensitized to MEK inhibition.

Subsequently, we asked whether the production of bFGF in the resistant cells was dependent on either ERK1/2 and mTOR activity or the expression of G1-regulators that we had shown to mediate palbociclib resistance (Figure 6I). Using qRT-PCR, we determined that treatment with PD0325901 or everolimus for 48 hours, but not the AKT inhibitor MK2206, blocked the increase in bFGF transcripts observed in H358-PR250 cells. In contrast to the effects of PD0325901 and everolimus, MK2206 treatment caused a substantial increase in bFGF expression in parental H358 cells. Additionally, the depletion of CDK6, cyclin D1 or p27^Kip1^ also diminished bFGF levels. Depletion of cyclin D3 was shown to have no effect on bFGF mRNA expression.

### Palbociclib resistance *in vivo* recapitulates findings in cell lines and is overcome by concomitant MEK inhibitor treatment

**Figure 7:**
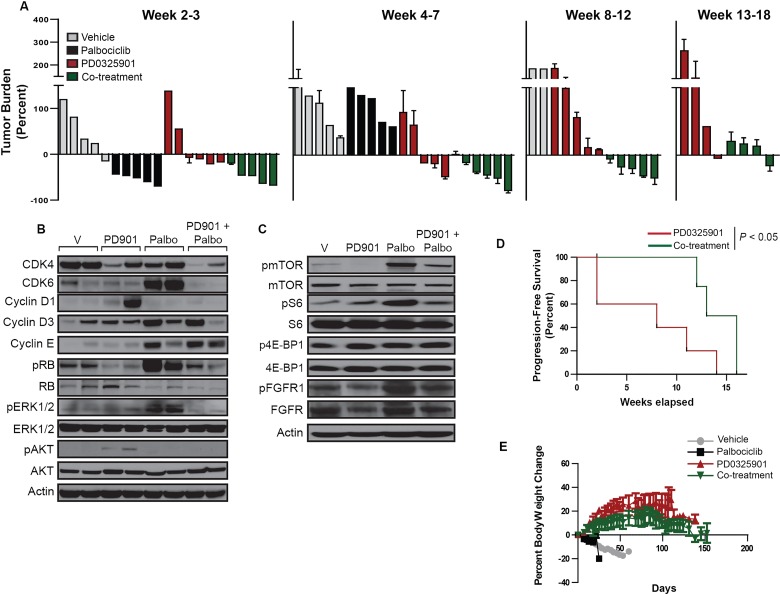
The palbociclib-PD0325901 combination reduces tumor burden and improves progression-free survival in a genetically-engineered mouse model of *KRAS*-mutant NSCLC **A.** Waterfall plot indicating the percent change in tumor burden of mice treated with vehicle, palbociclib alone, PD0325901 alone or the palbociclib-PD0325901 combination for up to 18 weeks. **B.** Western blot analysis of CDK and cyclin expression and ERK and AKT activation in tumor lysates isolated at the study endpoint for each treatment group. **C.** Western blot analysis of the activity of the mTOR pathway in tumor lysates isolated at the study endpoint for each treatment group. **D.** Kaplan-Meier survival plots for experimental groups treated with PDO325901 alone or in combination with palbociclib. Significance was assessed using the Log rank test for trend. **E.** Percent body weight change for mice in the 4 treatment groups over the treatment duration.

To model CDK4/6 inhibitor resistance *in vivo*, we assessed the efficacy of palbociclib in a genetically-engineered mouse model (GEMM) of *Kras-*mutant NSCLC. As shown in Figure 7A, an initial therapeutic response to palbociclib alone was observed (weeks 2-3) that was lost by week 4 of treatment. We also assessed signaling and cell cycle proteins in tumor samples collected at the study endpoint, i.e. at the time of acquired palbociclib resistance (Figure 7B and 7C). Compared to tumors isolated from vehicle-treated animals, those from palbociclib-treated animals demonstrated increased expression of CDK6, cyclin E and increased RB phosphorylation. One of the two tumors assessed also had increased expression of cyclin D3. Additionally, increased activation of ERK1/2, mTOR, and S6, as well as increased expression and activation of FGFR1, were all demonstrated in palbociclib- resistant tumors, confirming findings demonstrated in *KRAS*-mutant NSCLC cell lines.

We next determined whether resistance to palbociclib could be prevented or delayed with concomitant MEK inhibition. Among animals treated with PD0325901 alone, a response was seen in a subset that was maintained through the 18-week period (Figure 7A). Nonetheless, the majority of animals in this group completed the study with a high tumor burden. In contrast, all animals treated with the combination of palbociclib and PD0325901 demonstrated a response that was maintained through the duration of the study, with only a slight increase in tumor burden observed near study completion (Figure 7A). While overall survival of the PD0325901 alone and palbociclib/PD0325901 groups was similar (data not shown), the group treated with the combination was shown to have a significant improvement in progression-free survival (Figure 7D), with stable body weight (Figure 7E).

As shown in Figure 7B, compared to tumors isolated from vehicle-treated animals, tumors from animals treated with PD0325901 alone were shown to have increased AKT activation; one of the 2 tumors assessed also had increased cyclin D1 expression.

Compared to tumors isolated from animals treated with palbociclib alone, tumors isolated from animals treated with the palbociclib/PD0325901 combination demonstrated similarly increased expression of cyclins D3 and E; however, levels of CDK6, pERK, activated mTOR and S6, as well as of activated FGFR1, were similar to those observed in vehicle-treated animals (Figure 7B and 7C). Moreover, the increased expression of cyclin D1 and activation of AKT, observed in the PD0325901 monotherapy group, were also not observed in animals treated with the combination. These results suggest that the combination represses the emergence of resistance mechanisms that operate after palbociclib or PD0325901 monotherapies.

## DISCUSSION

CDK4/6 inhibition is being actively investigated as a therapeutic strategy in NSCLC and has produced clinical benefit among patients with tumors harboring *KRAS* mutation [14]. It is therefore essential to understand mechanisms that mediate sensitivity and resistance. Although RB loss has been identified as a palbociclib resistance mechanism [26–29], RB expression and phosphorylation were maintained in our palbociclib-resistant cell lines. Both *CDK6* and *CCNE1* amplification have also been implicated in palbociclib resistance [30, 31]. We observed a significant increase in both CDK6 and cyclin E expression in palbociclib-resistant *KRAS*-mutant NSCLC cells, as well as increased expression of D-cyclins. Increased expression of G1 cell cycle proteins was reversible when the selective pressure of CDK4/6 inhibition was removed, suggesting these changes were not due to genomic amplification. Expression of CDK6 and D-type cyclins was linked to activation of MEK-ERK signaling, so that palbociclib-resistant cells demonstrated dependence on the MAP kinase pathway with enhanced sensitivity to MEK and ERK inhibition.

Recently, in neuroblastoma models, increased ERK activation was associated with insensitivity to the CDK4/6 inhibitor ribociclib [32]. In palbociclib-resistant *KRAS*-mutant NSCLC cells, ERK-dependent (but not AKT-dependent) phosphorylation of TSC2 blocked its ability to negatively regulate the mTOR pathway, resulting in mTOR activation. Increased signaling through mTOR was associated with palbociclib resistance and resistant cells became sensitized to mTOR inhibition, as with MEK and ERK inhibition. In palbociclib-resistant cells, both MEK and mTOR inhibition resulted in reduction of cyclin D-CDK6 activity, as well as cyclin E-CDK2 activity, the latter likely via modulation of the expression and distribution of p27^Kip1^.

Interestingly, CDK6 has also been demonstrated to a mediate the phosphorylation of TSC2, with up-regulation of mTOR activity. Indeed, CDK4/6 inhibition was associated with a reduction in TSC2 phosphorylation in HER2-positive breast cancer mouse models and was shown to resensitize tumors to EGFR/HER2 blockade [33]. Therefore, the mechanism by which increased ERK activity observed in palbociclib-resistant cells may promote mTOR activation may be twofold: via the direct ERK-dependent phosphorylation of TSC2 or via the induction of CDK6 expression, a known regulator of TSC2 phosphorylation.

Our results have also identified increased FGFR1 activity as a key mediator of palbociclib resistance in *KRAS*-mutant NSCLC, which was due to secretion of bFGF and linked to elevated ERK and mTOR signaling, as well as increased expression of CDK6 and D-type cyclins. Interestingly, H358 cells have been shown to have low bFGF expression [34]; therefore, increased bFGF-driven signaling is highly specific to the resistant cell population. Of note, FGFR1 activity has also been implicated in MEK inhibitor resistance in *KRAS*-mutant NSCLC, where it activated MEK and AKT in MEK inhibitor-resistant cells, resulting in induction of epithelial-mesenchymal transition [35, 36]. Interestingly, our results also demonstrate that elevated signals through ERK1/2 and mTOR, as well as increased CDK6, cyclin D1 and p27^Kip1^ mediate the expression of the bFGF ligand within palbociclib-resistant cells. Notably, both cyclin D1 and CDK6 have been shown to play roles in cellular transcription independent of associated kinase activity [37, 38] Additionally, a D-cyclin-p27^Kip1^-CDK6 holoenzyme would be expected to increase cellular E2F activity, which has been linked to increased expression of both bFGF and FGFR1 [39, 40].

**Figure 8:**
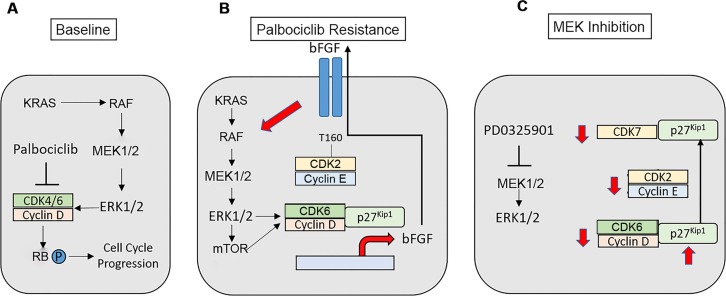
Modeling of palbociclib resistance in *KRAS*-mutant NSCLC cells and effects of MAP kinase pathway inhibition **A.** *KRAS*-mutant NSCLCs signal via the MAP kinase pathway to stimulate cell cycle progression. **B.** A bFGF autocrine/paracrine loop propagates and maintains palbociclib resistance in NSCLC. Increased signals through MEK-ERK1/2-mTOR enhance the expression, assembly and activity of cyclin D-CDK6, which promotes palbociclib-resistant proliferation and growth and enhances the transcription of the FGFR ligand bFGF. bFGF is secreted, where its activates its receptor to further enhance MEK-ERK1/2 signaling within the individual resistant cell as well as in neighboring cells. Increased MAP kinase activity in the palbociclib-resistant state also leads to increased cyclin E-CDK2 activity. **C.** MAP kinase pathway inhibition is expected to disrupt the autocrine loop, reducing the expression of D-cyclins and CDK6. MEK inhibition causes increased expression of p27^Kip1^, as well as a redistribution of p27^Kip1^ from CDK4/6 to CDK7 with maintenance of the association of p27^Kip1^ with CDK2. These events block the activating phosphorylation of CDK2 mediated by CAK, and directly block CDK2 activity. Therefore, MAP kinase inhibition reduces the activity of all G1 CDK complexes in palbociclib-resistant cells.

These results suggest that palbociclib-resistant cells establish a bFGF autocrine-paracrine loop that could promote the spreading of resistance to neighboring cells and the maintenance of resistance in each individual cell (Figure 8A and 8B). Additionally, our data indicate that MEK inhibition results in a redistribution of p27^Kip1^ to CDK7, reducing CAK activity and the activating phosphorylation of CDK2, so that increased cyclin E-CDK2 activity contributing to palbociclib resistance is also abrogated (Figure 8C). These results imply that Inhibition of FGFR, the MAP kinase pathway and mTOR would all be expected to disrupt the bFGF-driven autocrine loop. It will be important to compare the relative efficacy and tolerability of these approaches in *in vivo* preclinical models in order to facilitate translation to the most promising clinical trials.

The biologic outcome of CDK4/6 inhibition is not limited to G1 arrest and quiescence, but also may involve induction of senescence. The conversion of reversible G1 arrest to a senescent phenotype in response to CDK4/6 inhibition occurs in a subset of cell lines and primary cancers and has been linked to downregulation of the MDM2 protein, dependent on its E3 ligase activity, as well as on the presence of ATRX, which likely converge on expression and stabilization of a senescence-activating protein [41]. Interestingly, H358 cells have been shown not to downregulate MDM2 following CDK4/6 inhibition and are therefore representative of cells that only achieve reversible quiescence [42]. The failure of these cells to undergo senescence may facilitate escape via upregulation of an RTK-dependent pathway. FGFR-mediated pathways may be relevant not only to NSCLC cells but also to breast cancer cells with acquired palbociclib resistance [43]. Conversely, however, high baseline activation of an RTK-dependent pathway (e.g. an IGF1R-AKT axis in liposarcoma cells) does not preclude induction of senescence following CDK4/6 inhibitor monotherapy [41, 44], and ultimately, mechanisms of CDK4/6 inhibitor resistance may be distinct in cells capable of achieving the senescent phenotype. Importantly, the effects of CDK4/6 inhibition in the NSCLC cell lines utilized here were reflected in the genetically-engineered mouse model of *Kras*-mutant lung adenocarcinoma. Palbociclib similarly did not induce senescence in this model [12] and resulted in FGFR1 pathway activation.

In addition to quiescence and/or senescence, CDK4/6 inhibition may also induce a proapoptotic transcriptional program by augmenting expression of SMAC and caspase-3 while simultaneously suppressing expression of IAPs, FOXM1 and survivin. These events have been described in H460 cells, as well as in other NSCLC cells lines, and occur in an RB-dependent manner [45]. The restoration of RB hyperphosphorylation with consequent inactivation by the mechanisms delineated in our work would therefore be expected to overcome both quiescence as well as a pro-apoptotic proclivity.

In *NRAS*-mutant melanoma models, it has been proposed that MEK inhibitor-dependent induction of apoptosis is countered by proliferative responses within the tumor. CDK4 was identified as a key mediator of this proliferation [15], such that combined CDK4 and MEK inhibition led to tumor regression similar to that achieved by NRAS extinction. These results have translated to a clinical trial, where combined ribociclib and binimetinib has produced responses in patients with *NRAS* mutant melanoma [46]. Combinations of CDK4/6 and MEK inhibitors have also been shown to be synergistic in preclinical models of pancreatic, colorectal cancer and lung cancer, and several additional clinical trials evaluating CDK4/6 inhibitors with either MEK or ERK inhibitors are ongoing (NCT02022982, NCT02703571). Here, we have demonstrated the combinatorial efficacy of CDK4/6 and MEK inhibition in an *in vivo* model of KRAS-driven NSCLC and have demonstrated another possible mechanism for the synergy, as palbociclib treatment selects for cells that acquire MEK-ERK-mTOR dependence. Of note, however, combined CDK4/6 and MAP kinase inhibition is also complicated by resistance, and in the case of melanomas, related to pS6 expression via PI3K pathway activation [47, 48], so that mTOR inhibition may be germane to reversing resistance to CDK4/6 inhibitor monotherapy, as well as combined CDK4/6 and MAP kinase inhibition. This points to the potential importance of developing combinations in which both CDK4/6 and mTOR are inhibited.

Other mechanisms for the combinatorial effects of CDK4/6 and MEK inhibition in the KRAS-driven lung adenocarcinoma model may also be contributing, including an induction of senescence not observed after exposure to palbociclib alone. In fact, RAS pathway downregulation has been shown to promote conversion of quiescence to senescence [42]. Further work will be required to determine the contribution of senescence to the improved progression-free interval observed in mice receiving combination treatment.

In summary, the present study highlights a novel mechanism of palbociclib resistance in *KRAS*-mutant NSCLC that stems from the ERK-dependent induction of CDK6 and cyclins D1 and D3, as well as increased expression of cyclin E. Increased expression of G1 cyclin-CDK complexes results in part from increased FGFR1 mediated-signaling that is transferable and maintained via bFGF secretion and that causes activation and dependence on a MEK-ERK-mTOR pathway. Consequently, palbociclib-resistant cells are sensitized to inhibitors of FGFR, MEK, ERK and mTOR. Inhibition of this pathway leads to palbociclib re-sensitization, with reduction in G1 cyclin-CDK activity, associated with reduced cell cycle progression and the induction of apoptosis. Therefore, combinatorial inhibition of CDK4/6 with pharmacologically targetable mediators of resistance may be a promising strategy in the treatment of *KRAS-*mutant NSCLC.

## MATERIALS AND METHODS

### Cell culture and drug treatments

*KRAS*-mutant NSCLC cell lines were authenticated and maintained in the recommended medium. Palbociclib (PD0332991) and PD0325901 were supplied by Pfizer. Trametinib, binimetinib, ulixertinib, everolimus, MK2206, erlotinib, LY2874455 and LMN-211904 were obtained from Selleckchem, and bFGF neutralizing antibody was obtained from Sigma. For depletion experiments, pLKO.1 shRNA constructs targeting CDK1, CDK2, CDK4, CDK6, cyclin D1, cyclin D3 and cyclin E (Harvard PlasmID Database) were expressed by lentiviral infection. Palbociclib-resistant cells H358-PR100, H358-PR250, H460-PR500 and H441-PR500 were isolated after the serial addition of increasing concentrations of palbociclib to culture media. Resistance was confirmed by assessment of proliferation and cell cycle analysis in the presence of palbociclib. Cell viability assays were performed using CellTiter-Glo (Promega) starting with 1 × 10^3^ cells in 96-well plates.

### Western blot analysis

Whole-cell lysates were prepared in RIPA Lysis buffer (Boston Bioproducts), containing protease and phosphatase inhibitors. Protein concentration was determined by BCA protein assay (Pierce) and equal amounts (25 μg) were subjected to SDS-PAGE. Western blot analysis was performed using monoclonal antibodies against CDK1, CDK4, CDK6, cyclin E (Santa Cruz Biotechnology) Cyclin D3, total S6 ribosomal protein, total RB, p27^Kip1^ (Cell Signaling Technology) and actin (Genscript) and polyclonal antibodies against CDK2, CDK4, p21^Waf1/Cip1^, pAKT, RSK1/2 (Santa Cruz Biotechnology), Cyclin D1, phospho-RB S807/811, phospho-RB T821, phospho-ERK1/2, total ERK1/2, total AKT, phospho-S6 ribosomal protein, phospho-4E-BP1, total 4E-BP1, total TSC2, phospho-TSC T1462, C-Raf, phospho-C-Raf, MEK1/2, phospho-MEK1/2, phospho-RSK1, E-cadherin, vimentin, N-cadherin, CDK7, phosphor-CDK2 T160 (Cell Signaling Technology) and phospho-TSC S1798 (Abgent).

### Tyrosine kinase and growth factor arrays

Tyrosine kinase and growth factor arrays (Raybiotech) were implemented as described in product protocols. Lysates from proliferating H358 and H358-PR250 cells were used for the tyrosine kinase array, whereas media obtained from confluent H358 and H358-PR250 monolayers was used in the growth factor arrays.

### Colony formation assay

Cells were seeded in 6-well plates at a concentration ranging from 100-1000 cells and allowed to grow out in the presence of indicated concentrations of drug for a period of 10-14 days. Colonies were fixed (3:1: methanol: acetic acid), rinsed and stained with 0.4% crystal violet. Colony number was assessed using Image J (NIH).

### Flow cytometry analysis

For cell cycle analysis, fixed cells were stained with propidium iodide (PI) and were assessed for DNA content using CellQuest software (BD Biosciences). Cell cycle distribution was determined using Modfit (Verity Software House). Apoptotic cells were detected using the FITC Annexin V Apoptosis Detection Kit I (BD Biosciences).

### CDK kinase assay

CDK2, CDK4 or CDK6 were immunoprecipitated from lysates obtained from H358 or H358-PR250 cells treated with DMSO or PD0925901 (100 nM) for 24 hours. Immunoprecipitates were washed in Kinase buffer [Cell Signaling, 25 mM Tris-HCl (pH 7.5), 5 mM -glycero-phosphate, 2 mM DTT, 0.1 mM Na_3_VO_4_, 10 mM MgCl2], incubated with 2 μg RB protein and 1 mM ATPγS in 30 μl of Kinase buffer for 30 minutes at 30°C, and then alkylated with 15 mM p-Nitrobenzyl mesylate for 2 hours at room temperature. Kinase activity was assessed by Western blot analysis using a monoclonal antibody to thio-phosphate ester (Abcam).

### RNA isolation and RT-PCR

RNA was isolated from H358 and H358-PR250 cells by Trizol extraction. RNA purity and concentration were assessed by Nanodrop. Reverse transcription was completed using Superscript Vilo (Life Technologies) as per the manufacturer's instructions. qPCR reactions were completed on a 7500 Fast Real-Time PCR System (Applied Biosystems) using the Taqman^®^ Gene Expression Master Mix (Life Technologies) and the following Taqman^®^ probes for p27^Kip1^: Hs00153277_m1 and bFGF: Hs00266645_m1.

### Animal treatments and assessments

Mice bearing lung adenocarcinomas driven by conditionally activatable *Kras^G12D^* were treated with 5 X10^6^ p.f.u. adeno-Cre intranasally as previously described [49]. Mice were administered 100 mg/kg palbociclib [50] and 1.5 mg/kg PD0325901 [51] daily by oral gavage and bi-weekly tumor burden was assessed by MRI [52]. MRI quantification was performed as described previously. All mice were housed and treated in accordance with protocols approved by the Dana-Farber Cancer Institute Animal Care and Use Committee.

### Statistical analysis

A one-way or two-way ANOVA with Bonferroni post-analysis or a Logrank test for trends were used to assess significance. *P* < 0.05 was considered statistically significant.

## SUPPLEMENTARY MATERIALS FIGURES AND TABLE




