# Supplementary material for: Palbociclib resistance confers dependence on an FGFR-MAP kinase-mTOR-driven pathway in KRAS-mutant non-small cell lung cancer
Source: Oncotarget. 2018 Aug 3;9(60):31572–89. doi: 10.18632/oncotarget.25803 (PMC6114982; doi:10.18632/oncotarget.25803)
Supplement: Supplementary file 1 [file oncotarget-09-31572-s001.pdf]

Palbociclib resistance confers dependence on an FGFR-MAP kinase-mTOR-driven pathway in *KRAS*-mutant non-small cell lung cancer

SUPPLEMENTARY MATERIALS

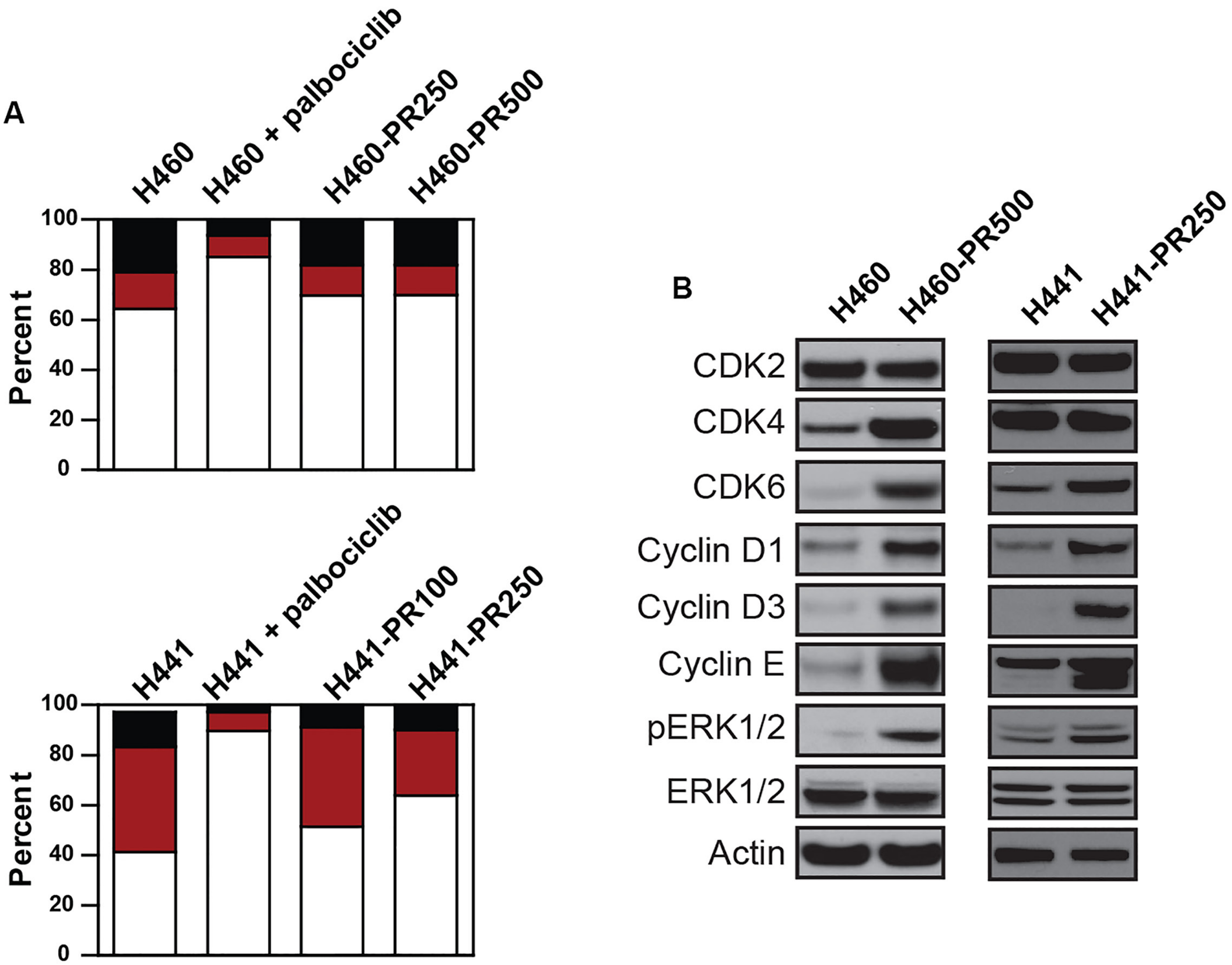

Supplementary Figure 1: Isolated palbociclib-resistant H441 and H460 cell lines are also sensitized to MEK inhibition. **A.** Cell cycle profile of H441 and H460 cells treated with vehicle control or palbociclib (250 nM) for 24 hours and H441-PR100, H441-PR250, H460-PR250 and H460-PR500 resistant clones grown in the presence of palbociclib. **B.** Assessment of CDK and cyclin expression and ERK1/2 activity in H460, H460-PR500, H441 and H441-PR500 cells by Western blot analysis.

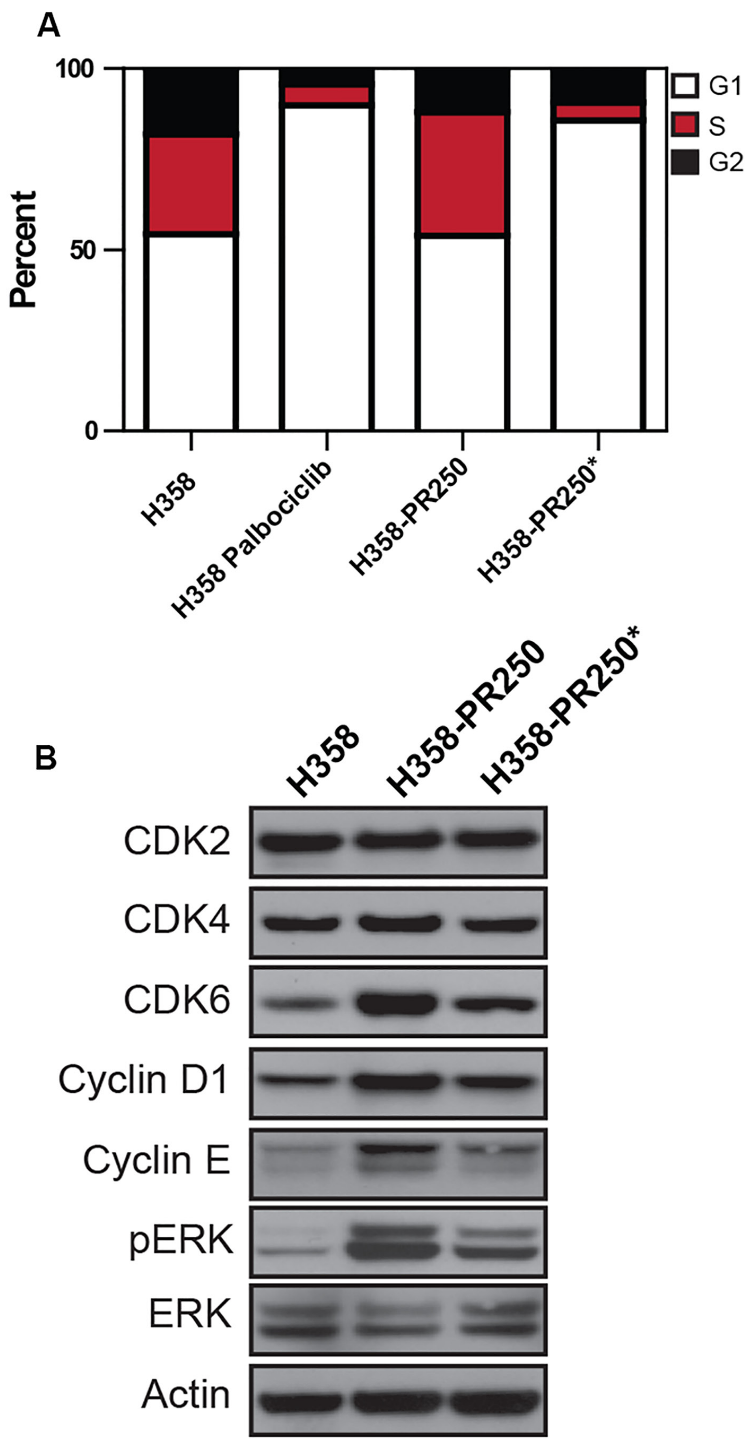

Supplementary Figure 2: Palbociclib-resistance is reversible in NSCLC cell lines. A. Cell cycle analysis of H358 cells treated with DMSO or palbociclib (250 nM) for 24 hours, H358-PR250 cells continuously grown in the presence of palbociclib (250 nM) and H358-PR250* cells grown in the absence of palbociclib for a period of 6 weeks then challenged with palbociclib (250 nM) for 24 hours. **B.** Western blot analysis assessing the expression of CDKs and cyclins and ERK1/2 activation in H358, H358-PR250 and H358-PR250*.

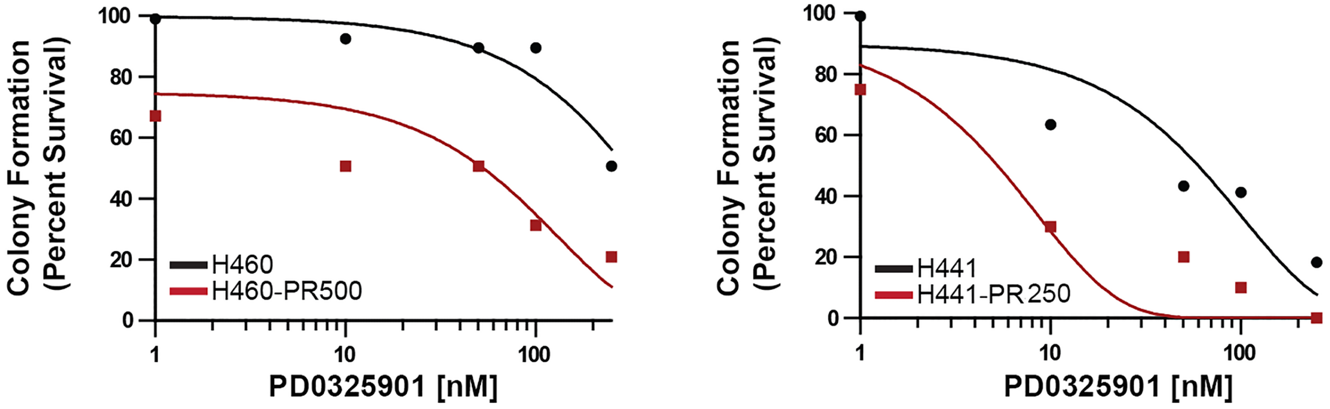

Supplementary Figure 3: Colony formation assay in H460 and H460-PR500 (*left panel*) or H441 and H441-PR500 (*right panel*) cells treated with the indicated doses of PD0325901 for 14 days.

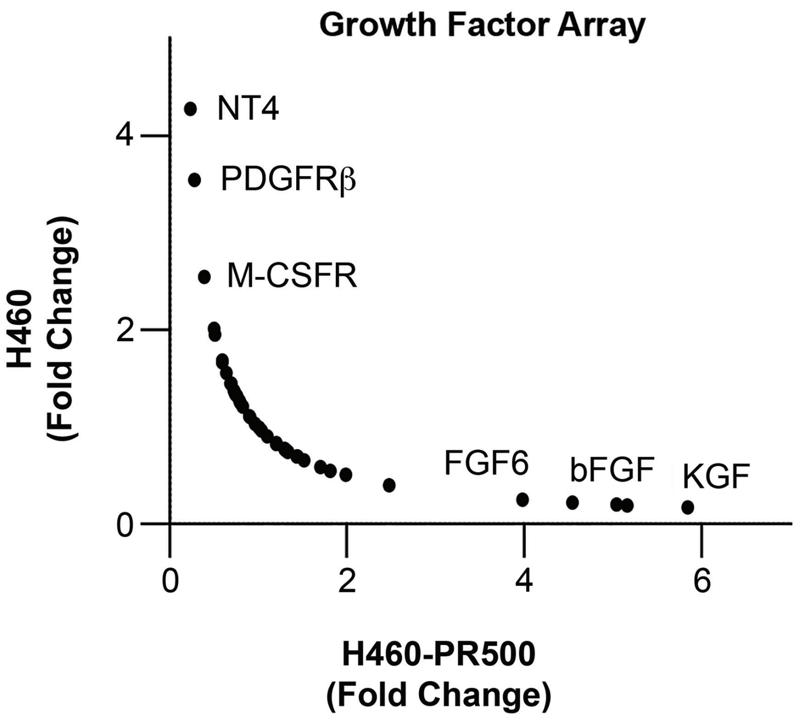

Supplementary Figure 4: bFGF secretion by palbociclib-resistant NSCLC cell line. Fold change in the secretion of growth factors by H460 cells grown in the absence of palbociclib and H460-PR500 cells grown continuously in palbociclib obtained from a growth factor array.

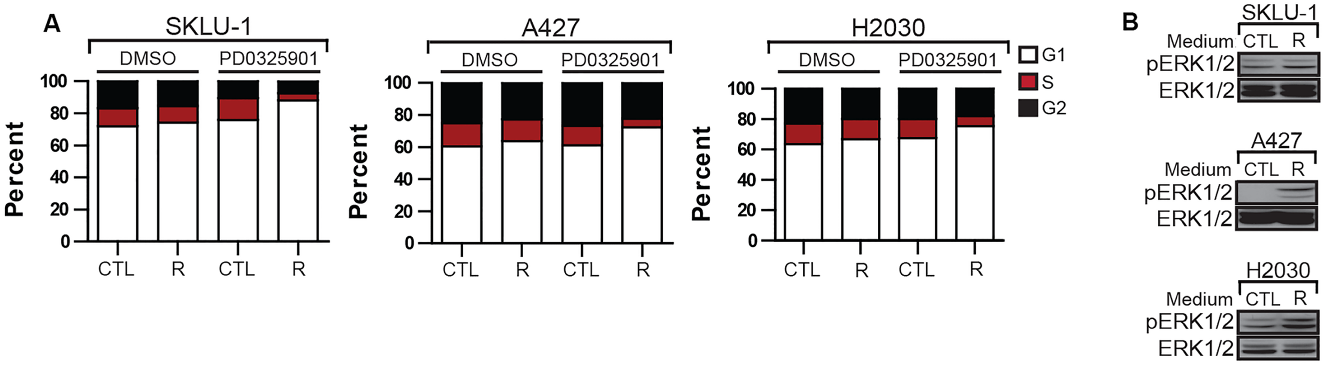

Supplementary Figure 5: MEK inhibitor sensitivity is transduced from one NSCLC cell line to another. A. Cell cycle profile of SKLU-1, A427 and H2030 cells grown in control (CTL) or resistant (R) medium for 72 hours. Medium was isolated from parental H358 and H358-PR250 cells, respectively. **B.** Western blots demonstrating activation of ERK1/2 in parental cells grown in control medium or medium isolated from H358-PR250 cells.

For Supplementary Table 1 see attached word file in Supplementary Files.
