# Supplementary material for: Palbociclib resistance confers dependence on an FGFR-MAP kinase-mTOR-driven pathway in KRAS-mutant non-small cell lung cancer
Source: Oncotarget. 2018 Aug 3;9(60):31572–89. doi: 10.18632/oncotarget.25803 (PMC6114982; doi:10.18632/oncotarget.25803)
Supplement: Supplementary file 2 [file oncotarget-09-31572-s002.docx]

**
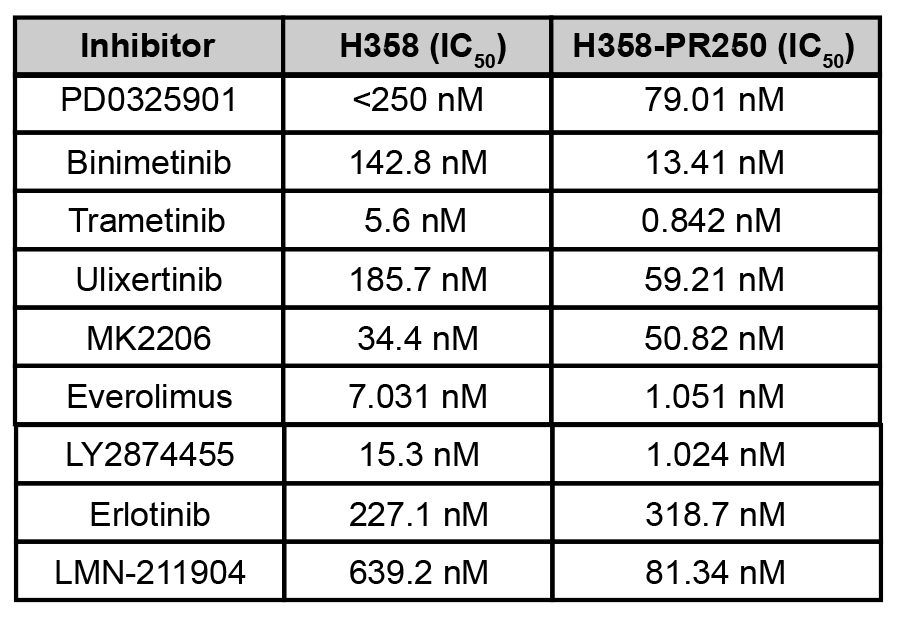
Supplementary Table 1**

**IC50 of compounds in palbociclib-sensitive and -resistant *KRAS*-mutant NSCLC cell lines.** IC_50_’s were determined from the mean cell survival of three independent experiments assessed by a colony formation assay. Drug exposure was for 14 days.
